# Effect of knee extensor fatigue level and sex on bilateral jump-landing

**DOI:** 10.1136/bmjsem-2019-000660

**Published:** 2020-01-27

**Authors:** Byungjoo Noh, Chang Hong Youm, Myeounggon Lee, Hwayoung Park, Minji Son, Jinhee Kim

**Affiliations:** 1 Department of Health Care and Science, College of Health Sciences, Dong-A University, Saha-gu, Busan, South Korea; 2 Biomechanics Laboratory, College of Health Sciences, Dong-A University, Saha-gu, Busan, South Korea

**Keywords:** muscle fatigue, jump-landing, biomechanics, injury

## Abstract

**Objective:**

The purpose of this study was to determine the effect of fatigue level and sex on the range of motions of the lower extremities and impulses during the jump-landing phase after performing bilateral fatiguing tasks.

**Methods:**

In total, 41 healthy young adults volunteered for this study. Participants’ jump-landing trajectories were monitored using nine cameras, and ground reaction forces were measured using a force plate. Participants performed five maximal bilateral countermovement jumps as prefatiguing tasks. The fatiguing tasks consisted of maximal effort contractions of the knee extensor at 60°/s on a dynamometer until task failure, defined as the inability to reach 50% of the peak knee extension torque for three consecutive times. The post-task maximal bilateral jumps were immediately captured after the participants failed the fatiguing task. Participants were asked to perform this cycle again, performing the fatiguing contraction task until failure to reach 30% of the peak knee extension torque.

**Results and conclusion:**

It was found that the knee joint was more extended in the post-30% fatiguing task, which was due to the reduction of the flexion angle of the hip and knee joints in response to fatigue level. The impulses for both sexes were reduced at the severe fatigue level. Fatigability altered jump-landing kinematics, jump heights and impulses in response to fatigue level. The post-30% fatiguing task elicited more fatigue than the post-50% fatiguing task.

**What are the new findings?**

- Vertical jump height was related to fatigue level in both sexes.
- Extension of the knee joint occurred in the 30% fatiguing task, and the flexion angle of the hip and knee joints was reduced in response to muscle fatigue level.
- Reduction of impulses occurred at severe fatigue levels for both sexes.
- Fatigability was altered in response to fatigue level, with the post-30% fatiguing task generating more obvious fatigue than the post-50% task.

## Introduction

Jumping and landing movements are major skill components in sports as well as in human movement.^1^ These movements can provide an objective indication of maximal exercise capacity as well as the agility of the lower extremities.^2^ Jumping is a simple way to raise the centre of mass as high as possible using the exerted reaction force from the ground on the human body.^3^ The accompanying landing movement is associated with musculoskeletal injuries, especially in the lower extremities.^4^

Muscle fatigue can be defined as an exercise-induced temporary reduction in the force and power production of the skeletal muscles.^5^ Muscle fatigue can occur in response to impaired muscle function, called peripheral fatigue, and a reduction in the neural drive from the central nervous system to the activated muscles, called central fatigue.^5–7^ Muscle fatigue can contribute to musculoskeletal injury, as well as reduce the time and energy efficiency in playing sports.^8 9^ Thus, several muscle fatigue studies have been conducted to determine associated injuries and to enhance sports performance during jumping and landing. Hunter^10^ showed that muscle fatigue differs between sexes, with women having a relatively longer duration before reaching muscle fatigue via the peripheral fatigue mechanism after performing both maximal and submaximal isometric tasks.

Fundamentally, jump-landing performance is influenced by kinetic, kinematic and physical parameters, in addition to muscle fatigue.^11^ However, it is unknown whether kinetic and kinematic differences due to fatigue level or sex occur during jump-landing when bilateral fatigue is generated. The purpose of this study was to determine the effects of fatigue level and sex on the kinematic variables and impulses during jump-landing after bilateral fatiguing tasks. We hypothesised that vertical jump height, range of motions of the lower extremities and impulses when jump-landing would be different depending on the sex and muscle fatigue level of the subject. We also suspected that fatigability would be different in response to fatigue level.

## Methods

### Participants

Forty-one healthy young adults who exercise at least 3 days a week (21 men, age: 20.5±0.9 years; 20 women, age: 21.4±1.9 years) volunteered. Participants were excluded from the study if they had musculoskeletal injuries or surgery on their lower extremities within the last 6 months.

### Data collection

The marker trajectories data captured jump-landing using the three-dimensional motion analysis system of nine cameras (Vicon MX-T10; Oxford Metrics, Oxford, UK), with a sampling frequency of 100 Hz. The ground reaction force (GRF) was collected with a force plate (OR6-7, AMTI, Watertown, Massachusetts) at 2000 Hz. The marker trajectories data were captured using the Vicon Nexus software package (Oxford Metrics). Twenty-six retroreflective markers (with a diameter of 14.0 mm) were placed according to the modified Plug-in Gait model of the Helen Hayes marker set.^12^ To reduce motion artefacts, all markers were secured with double-sided tape and Kinesio tape.

This study was split into two sessions, with a 7-day interval between sessions. All participants attended a familiarisation session that involved biometric tests, measurement of the lower extremity joint centre and knee extension peak torque, and practice of the jump and landing. The participant’s dominant leg (ie, the leg used to kick a ball) was placed on the force plate, and then they performed the maximal bilateral countermovement jump with their hands on the hips and then landing back on the force plate.^13^ To test the knee extension peak torque, each participant was seated with the hip and knee angles at 85° and 90°, respectively, on a dynamometer (Cybex Humac Norm, CSMI, Stoughton, Massachusetts). Both lower legs were strapped to the distal end of the dynamometer arm, with the lateral epicondyle of the femur aligned with the axis of rotation of the dynamometer.

### Knee extensor fatiguing contraction task

Participants rode a stationary bike at their preferred speed for 10 min and practised the jumps for a few times as a warm-up exercise for the lower extremities in order to reduce the risk of injury. Following this warm-up, the prefatiguing task, involving five maximal bilateral countermovement jumps, was captured prior to starting the fatiguing contraction task. Participants then moved to the dynamometer to perform the fatiguing contraction task, which was similar to the method of testing the knee extension peak torque. Participants were asked to maximally extend their legs on the dynamometer to the full range of motion (0°–90°). The lower leg was then passively returned to the start position of 90° of knee flexion. The participants performed maximal effort contractions of the knee extensor at 60°/s until they failed to reach 50% of the previously measured peak knee extension torque for three consecutive times.^14^ After conducting the 50% fatiguing task, five maximal bilateral countermovement jumps were immediately conducted and captured (post-50% fatiguing task). Participants were asked to perform this cycle again, performing the fatiguing contraction until failure for three consecutive times to reach 30% of the peak knee extension torque and the maximal jumps five times (post-30% fatiguing task). Among the five jumps and between the jump testing and fatiguing contraction task, participants conducted jump testing as soon as possible in an effort to minimise fatigue recovery after the fatiguing contraction task.^14^

### Data analysis

Jump-landing data were interpreted as the average values of three successful trials in which participants landed on the force plate correctly from five bilateral jump-landing trials. Analysis of the jump-landing phase was carried out prior to the commencement of the fatiguing task to provide vertical jump heights as a baseline measurement for comparisons. The vertical jump heights were calculated by subtracting the height of the pelvis centre of mass during the static position from the maximum height during the jump. This measurement was repeated after failure at 50% and 30% of peak torque was observed in the fatiguing task. The joint angles were calculated based on the Euler/Cardan angles in the flexion/extension, adduction/abduction, and internal/external rotation order, namely y-x-z-axis rotation sequence. The kinematic and impulse data were filtered with a second-order Butterworth low-pass filter with a cut-off frequency of 10 Hz and 25 Hz, respectively. Data were analysed from the initial contact to the maximum flexion angle at the ankle, knee and hip joints of landing. The initial contact was defined as the moment when >10 N of vertical GRF (Fz value) was exerted by foot contact on the force plate.

### Statistical analysis

Statistical analysis was conducted using IBM SPSS Statistics V.20. Results are expressed as mean±SD. After confirmation of normality using the Shapiro-Wilk test, we performed a two-way repeated measures analysis of variance to evaluate fatigue level (pre, 50% and 30%) and sex. For the post-hoc analysis, the difference between fatigue levels was evaluated using a one-way analysis of variance, with the p value adjusted for multiple comparisons, using the Bonferroni method, to 0.0167 (0.05/3). The difference between male and female participants was evaluated using an independent t-test analysis. Values of p<0.05 were considered significant.

## Results

### Baseline results

There was no difference between men and women in the knee extension peak torque normalised to body mass (men, 6.29±1.73 Nm/kg; women, 6.29±1.83 Nm/kg, where t_39_=0.010, p>0.05). In addition, there was no sex-related or fatigue-related difference in the number of repetitions compared with baseline measurements (50% of peak knee extension torque: men, 39.6±8.1 Nm/kg vs women, 36.0±6.0 Nm/kg, where t_39_=1.612, p>0.05; 30% of peak knee extension torque: men, 54.3±19.8 Nm/kg vs women, 53.6±17.8 Nm/kg, where t_39_=1.612, p>0.05) (table 1).

**Table 1:** Baseline measurements

	PET/BM (Nm/kg)	50% NORs (repetitions)	30% NORs (repetitions)
Male	6.29±1.73	39.6±8.1	54.3±19.8
Female	6.29±1.83	36.0±6.0	53.6±17.8
t value	0.010	1.612	0.124

All data are presented as mean±SD.

The t values are the results of the independent t-test between male and female participants.

30% NORs, number of repetitions before decreasing to less than 30% of the peak knee extension torque; 50% NORs, number of repetitions before decreasing to less than 50% of the peak knee extension torque; PET/BM, peak extension torque of the knee by body mass.

### Vertical jump height

Sex and fatigue level had a significant interactive effect on vertical jump height (*F*_2,78_=22.079, p<0.001), further to the main observed effects of sex (*F*_1,39_=53.471, p<0.001) and fatigue level (*F*_2,78_=61.940, p<0.001). Post-hoc analysis indicated that men had significantly higher jump heights than women at baseline as well as after the post-50% and post-30% fatiguing tasks (t_39_=9.278, p<0.001; t_39_=7.078, p<0.001; t_39_=4.687, p<0.001, respectively). Additionally, the post-50% and post-30% fatiguing task data revealed significantly decreased jump heights in women compared with baseline values (*F*_2,38_=8.490, p=0.001). The jump height in men also decreased significantly in the following order: baseline, post-50% and post-30% fatiguing task causing the greatest reduction in jump height (*F*_2,40_=58.896, p<0.001) (table 2).

**Table 2:** Results for vertical jump height (cm)

	Pre	Post-50%	Post-30%	*F* value†	*F* value‡	Post-hoc
Male	44.8±4.9	40.8±4.9	38.0±5.6	53.471* (S)	58.896*	1>2>3
Female	32.9±3.1	31.6±3.2	31.4±3.3	61.940* (F)	8.490*	1>2,3
t value	9.278*	7.078*	4.687*	22.079* (S×F)		

All data are presented as mean±SD.

The t values are the results of the independent t-test between male and female participants.

*P<0.05.

†*F* value, results of the two-way analysis of variance with repeated measures between sex (S) and fatigue level (F).

‡*F* value, results of the one-way analysis of variance with repeated measures within pre, post-50% and post-30% fatiguing tasks.

### Range of motions of the ankle joint during jump-landing

The main effects of fatigue level were on the ankle flexion angle (*F*_2,78_=3.276, p=0.043). Post-hoc analysis indicated a significantly smaller dorsiflexion angle during the post-30% fatiguing task than at baseline or during the post-50% fatiguing task in men (*F*_2,40_=6.794, p=0.003). Fatigue level had no significant effect on the ankle flexion angle in women. There were no interactive effects between sex and fatigue level, and no effect was observed for sex alone (table 3).

**Table 3:** Range of motions of the ankle, knee and hip joints during jump-landing (°)

			Pre	Post-50%	Post-30%	*F* value†	*F* value‡	Post-hoc
Ankle	Dorsiflexion (+)/plantarflexion (−)	Male	59.39±6.51	60.43±7.47	56.46±6.97	3.615 (S)	6.794*	2,1>3
		Female	61.53±6.30	62.91±4.96	62.39±7.83	3.276* (F)	0.525	ns
		t value	1.064	1.248	2.561*	2.840 (S×F)		
	Eversion (+)/inversion (−)	Male	4.23±2.14	4.19±1.98	4.18±1.94	0.983 (S)	0.033	ns
		Female	3.61±1.68	3.53±2.01	3.69±2.37	0.083 (F)	0.103	ns
		t value	1.018	1.058	0.731	0.098 (S×F)		
	Internal (+)/external rotation (−)	Male	22.22±6.78	22.12±6.76	21.55±6.74	1.208 (S)	0.332	ns
		Female	23.56±5.17	24.76±6.11	23.63±5.87	1.075 (F)	1.553	ns
		t value	0.712	1.308	1.054	0.608 (S×F)		
Knee	Flexion (+)/extension (−)	Male	45.80±9.57	48.07±9.53	44.17±9.46	5.437* (S)	3.130	2>3
		Female	52.86±11.86	55.65±10.92	49.98±9.84	9.266* (F)	6.453*	2>3
		t value	2.102*	2.371*	1.929	0.334 (S×F)		
	Adduction (+)/abduction (−)	Male	8.51±3.86	8.89±4.65	8.18±3.76	1.884 (S)	0.550	ns
		Female	9.89±4.31	10.63±5.19	10.81±6.82	0.583 (F)	0.790	ns
		t value	1.080	1.133	1.542	0.795 (S×F)		
	Internal (+)/external rotation (−)	Male	15.17±6.02	17.01±6.38	17.09±5.86	1.958 (S)	6.092*	3,2>1
		Female	13.54±3.76	14.17±4.41	15.04±3.88	9.475* (F)	4.287*	3>1
		t value	1.035	1.646	1.314	1.128 (S×F)		
Hip	Flexion (+)/extension (−)	Male	22.92±9.85	17.08±9.91	14.68±8.91	5.391* (S)	11.852*	1>2>3
		Female	30.10±14.20	26.01±11.76	20.67±11.66	25.480* (F)	14.313*	1>2>3
		t value	1.889	2.634*	1.856	0.708 (S×F)		
	Adduction (+)/abduction (−)	Male	4.66±1.82	4.47±1.70	4.38±1.71	0.043 (S)	0.219	ns
		Female	4.60±1.63	4.69±1.63	4.48±1.57	0.258 (F)	0.144	ns
		t value	0.102	0.407	0.192	0.111 (S×F)		
	Internal (+)/external rotation (−)	Male	8.92±2.69	7.52±3.17	6.17±2.87	0.034 (S)	9.782*	1>3
		Female	8.76±3.10	7.34±3.39	6.07±2.52	20.127^*^ (F)	10.473*	1>3
		t value	0.183	0.168	0.117	0.005 (S×F)		

All data are presented as mean±SD.

The t values are the results of the independent t-test between male and female participants.

*P<0.05.

†*F* value, results of the two-way analysis of variance with repeated measures between sex (S) and fatigue level (F).

‡*F* value, results of the one-way analysis of variance with repeated measures within pre, post-50% and post-30% fatiguing tasks.

ns, no significance.

### Range of motions of the knee joint during jump-landing

The main effects of sex and fatigue level were on the knee flexion angle (sex, *F*_1,39_=5.437, p=0.025; fatigue level, *F*_2,78_=9.266, p<0.001). Post-hoc analysis indicated that women had a significantly greater knee flexion angle than men at baseline and during the post-50% fatiguing task (baseline, t_39_=2.102, p=0.042; post-50%, t_39_=2.371, p=0.023). During fatigue, the knee flexion angle was significantly greater in the post-50% fatiguing task than at baseline and the post-30% fatiguing task in women (*F*_2,38_=6.453, p=0.004). Fatigue level had no significant effect on the knee flexion angle in men. There were no interactive effects between sex and fatigue level. Fatigue level had a significant effect on the knee rotation angle (*F*_2,78_=9.475, p<0.001). Post-hoc analysis indicated that significantly greater internal rotation occurred during the post-30% fatiguing task than at baseline for both sexes (men, *F*_2,40_=6.092, p=0.005; women, *F*_2,38_=4.287, p=0.021). However, there were no significant interactive effects between sex and fatigue level (table 3).

### Range of motions of the hip joint during jump-landing

The main effects of sex and fatigue level were on the hip flexion angle (sex, *F*_1,39_=5.391, p=0.026; fatigue level, *F*_2,78_=25.480, p<0.001). Post-hoc analysis indicated that women had a significantly greater hip flexion angle than men in the post-50% fatiguing task (t_39_=2.634, p=0.012). Hip flexion angles were significantly smaller in the following order in both sexes: post-30%, post-50 and baseline (men, *F*_2,40_=11.852, p<0.001; women, *F*_2,38_=14.313, p<0.001). However, there were no significant interactive effects between sex and fatigue level. The main effect of fatigue level on hip rotation angle (*F*_2,78_=20.127, p<0.001) was a significantly greater hip external rotation angle in both sexes during the post-30% fatiguing task compared with baseline (men, *F*_2,40_=9.782, p<0.001; women, *F*_2,38_=10.473, p<0.001). There were no significant interactive or main effects of sex on the hip rotation angle (table 3).

### Impulses during jump-landing

The impulses were analysed using vertical GRF (Fz value) that was normalised by body weight. The primary effects of fatigue level were observed in the jump-landing impulses (*F*_2,78_=7.916, p=0.001). Post-hoc analysis indicated that impulses during the post-30% fatiguing tasks were smaller than the baseline in men (*F*_2,40_=4.191, p=0.022). For women, jump-landing impulses were smaller during the post-30% fatiguing task than the post-50% fatiguing task (*F*_2,38_=3.759, p=0.032). However, sex and fatigue level had no significant interactive effect (table 4).

**Table 4:** Impulses during jump-landing

		Pre	Post-50%	Post-30%	*F* value†	*F* value‡	Post-hoc
Impulse (N/s)	Male	3.41±0.26	3.40±0.39	3.23±0.24	0.277 (S)	4.191*	1>3
	Female	3.48±0.68	3.47±0.44	3.28±0.52	7.916* (F)	3.759*	2>3
	t value	0.473	0.515	0.433	0.019 (S×F)		

All data are presented as mean±SD.

The t values are the results of the independent t-test between male and female participants.

*P<0.05.

†*F* value, results of the two-way analysis of variance with repeated measures between sex (S) and fatigue level (F).

‡*F* value, results of the one-way analysis of variance with repeated measures within pre, post-50% and post-30% fatiguing tasks.

## Discussion

This study demonstrates the differences between men and women in responses to fatigue level of the knee extensor during jump-landing. The following are the main findings of this study: (1) vertical jump height is related to fatigue level in both sexes; (2) extension of the knee joint occurs more in the post-30% fatiguing task, and reductions in the flexion angle of the hip and knee joint occur in response to muscle fatigue level; (3) a reduction of impulses occurs at severe fatigue levels for both sexes; and (4) jump-landing kinematics/impulses are altered in response to fatigue level, with the post-30% fatiguing task generating more obvious fatigue than the post-50% task. These findings are discussed in detail in the sections that follow.

### Baseline measures and sex differences

No differences were observed between men and women with regard to the number of repetitions required to reach failure in the fatiguing tasks. The fatiguing protocol used in the present study can be related to the bilateral limb deficit (BLD) phenomenon.^15–17^ BLD is due to an insufficient neural input from the unilateral limb; therefore, more low-threshold motor units tend to be recruited when both limbs are contracting, resulting in a decline in force.^18^ However, force production can be sustained for a longer period of time because low-threshold motor units are recruited from the initial contraction up to the terminal contraction.^19^ In addition, women have a larger number of type I muscle fibres (which incorporate low-threshold motor units) than men.^20^ Our fatiguing protocol was based around bilateral contraction, which recruits more low-threshold motor units. Thus, failure of the task can occur faster than during unilateral fatiguing protocols. This could be one of the reasons why no interactions between sex and fatigue level were observed. However, further research is needed to determine whether bilateral contraction results in faster task failure than unilateral contraction or to differences in time to task failure between sexes.

### Range of motion characteristics of the lower extremities during jump-landing

In both sexes, a more extended knee joint was apparent in the post-30% fatiguing task. This was due to a reduced change in the flexion angle of the hip and knee joints during landing in response to fatigue level. We also observed more extended hip and knee joints as well as decreased ankle range of motion during jump-landing according to fatigue level in men. This indicated a diminished shock absorption capacity in the more extended joints, which was dependent on sex and fatigue level as previously reported.^21^ The greater compressive impact load on the cartilage could afflict the soft tissues.^22^ Thus, this strategy was considered the effects of sex on knee joint control due to the quadriceps fatigue during jump-landing.

The internal rotation angles of the knee joint were significantly affected by fatigue level. Specifically, the results showed that the greater the fatigue, the greater the internal rotation angle of the knee joint, although there was no interaction between sex and fatigue level. Previous studies have reported that a greater internal rotation angle of the knee joint is a risk factor for knee injury.^23^ As the increased internal rotation angle of the knee is dependent on fatigue level, it may be due to a malfunction in neuromuscular control, such as in the latter half of a sports match/game.^4^ Knee injuries due to neuromuscular fatigue could, therefore, be linked to alterations in the landing mechanics of the lower extremities. Thus, we suggest that the greater internal rotation angle of the knee joint due to more severely fatigued muscles should be addressed as a primary joint kinematic factor for knee injuries.

We also found that the higher jump height in men was due to the greater peak torque of men in maximal voluntary contraction than in women.^24^ Furthermore, the jump height decreased following severe fatigue levels in both sexes. This may be linked to a stiff landing as mentioned above. Fundamentally, to absorb shock during jump-landing even though the jump height had decreased, a more flexed knee joint is required,^25^ whereas in our study men had more extended hip and knee joint during jump-landing. Thus, a higher jump height with a stiff landing in men might pose a relatively higher risk of soft tissue injury despite the diminished jump height following severe fatigue levels.

### Impulses during jump-landing

The impulses for both sexes were diminished following the fatiguing tasks compared with baseline, with the post-30% fatiguing task having the greatest effect. This leads us to suggest that severe fatigue levels are related to the recruitment threshold of motor units in the knee extensors. In a previous study, regression analysis of the average motor unit firing rate versus the recruitment threshold relationship showed that as the muscle became more fatigued, the y-intercept decreased and the slope became shallower.^26^ Motor unit firing rates have been reported to be inversely proportional to the recruitment threshold.^26^ As this study demonstrated, this could lead to earlier-recruited motor units and higher motor unit firing rates when the muscle is fatigued, known as the ‘onion skin’ scheme.^27 28^ This might occur due to the increased excitation of the motor neurons with greater firing variability.^29–32^ This effect compensates for changes in the mechanical characteristics of the muscles that occur during fatigue.^33^ As the post-30% fatiguing task elicited more fatigue, there was a bigger effect on the performance. Therefore, we speculated that this was related to decreased impulses during landing due to low-threshold motor units and increased motor unit firing rates at the severe fatigue level.

### Limitations

This study and its experimental design have a few limitations that should be considered when interpreting the results. First, some of the results, especially the rotation angle of the hip joint in the post-50% fatiguing task, were greater in the post-30% fatiguing task. We speculate that this could be due to a recovery in the force production between fatiguing tasks and moving to the jumping task, as previous studies have reported,^34^ despite efforts to minimise the time between tasks. Second, this study did not directly measure the motor firing behaviour because the lower extremity muscles do not possess surface electromyographic (EMG) signals. Moreover, this study measured the jump height to confirm the effects of fatigue instead of measuring the lower limb muscle activities using EMG. Third, only the dominant leg was placed on the force plate despite participants performing the bilateral countermovement jump. However, this should only result in minimal impact on the reliability of the data because muscle fatigue was elicited with both legs strapped to the dynamometer arm.

## Conclusion

This study investigated the range of motions of the lower extremities and impulse changes during jump-landing related to different bilateral fatigue levels and sex. The results of this study showed differences in the range of motion of the knee and hip joints, as well as the landing impulse, for both sexes and muscle fatigue levels. This indicates that fatigability altered jump-landing kinematics, jump heights and impulses for both sexes in response to fatigue level. The post-30% fatiguing task elicited more fatigue than the post-50% fatiguing task. This study may provide insights into preventing lower limb injuries due to high fatigue levels.
